# A chronic myeloid leukemia case with a variant translocation t(11;22) (q23;q11.2): masked Philadelphia or simple variant translocation?

**DOI:** 10.11604/pamj.2018.30.161.9318

**Published:** 2018-06-22

**Authors:** Kadir Acar, Burak Uz

**Affiliations:** 1 Gazi University Faculty of Medicine, Department of Internal Medicine, Division of Hematology, Turkey

**Keywords:** Cronic myeloid leukemia, variant translocation, masked Philedelphia chromosome, imatinib mesylate, nilotinib

## Abstract

Chronic myeloid leukemia (CML) is characterized by the presence of the Philadelphia chromosome (Ph), usually due to a reciprocal translocation, t(9;22)(q34;q11.2). The remaining cases (2-10%) have variant translocation, and more rarely (~1%) a cryptic rearrangement is present which can be detected by fluorescence in situ hybridization analysis in a CML patient with a Ph-negative karyotype (Masked Ph). We present a masked/variant BCL-ABL-positive CML patient showing a t(11;22)(q23;q11.2) which was detected using a combined approach of conventional cytogenetics and reverse transcription polymerase chain reaction. In February 2013, the patient was diagnosed as having CML. Imatinib mesylate (400 mg/day), was then started. Under imatinib therapy a complete hematologic and cytogenetic response was attained. In December 2013, an increment in BCR-ABL/ABL transcript levels according to the International Scale (from 0.0471% to 1.4034%), indicating imatinib failure, was documented. Administration of nilotinib (400 mg twice daily) resulted in durable molecular response after 3 months. The patient is still on nilotinib treatment throughout the observation period with no sign of recurrence and adverse events.

## Introduction

Chronic myeloid leukemia (CML) is a clonal myeloproliferative disease that originates from hematopoietic stem cells in the bone marrow. CML is characterized by the translocation t(9;22)(q34;q11.2) called Philadelphia (Ph) chromosome. This translocation transfers Abelson murine leukemia oncogene (ABL1) on chromosome 9q34 to breakpoint cluster region (BCR) on chromosome 22q11 leading to BCR/ABL1 gene fusion. This fusion gene encodes an oncogene (P210, more rarely P230 or P190) which produces an abnormal tyrosine kinase activity that causes aberrant myelopoiesis [1]. In 2-10% of newly diagnosed CML cases, one or more additional chromosomes are added to 9 and 22, and are involved in the translocation [2]. These translocations are termed as “variant translocations”. Two variant subgroups have traditionally been defined as simple and complex. In simple variant translocations; the 22q11 segment is translocated on chromosome other than 9. In complex variant translocations; there is a third translocation, t(9;22;V), where “V” is a variable partner chromosome [3]. Only in a few cases; a cryptic rearrangement is found which could not be detected by conventional cytogenetics, but FISH reveals the characteristic BCR/ABL1 fusion gene, called “masked Ph” [4]. We would like to share our experience of a masked/variant BCR-ABL-positive CML patient showing a t(11;22)(q23;q11.2) who is successfully treated with tyrosine kinase inhibitors (TKIs).

## Patient and observation

A 31-year-old male presented with spontaneous ecchymosis on his right forearm and left lomber region, and epistaxis of 1 week duration. Physical examination was nonremarkable except for the gross splenomegaly (12 cm palpable under the left costal margin) and ecchymotic areas. Blood tests revealed leukocytosis (160 x109/L), thrombocytopenia (96 x109/L), and mild anemia (12.7 g/dL). Peripheral blood smear consists of 4% promyelocytes, 20% myelocytes, 30% metamyelocytes, 40% neutrophils, 2% eosinophils, 3% bazophils, and 1% normoblasts. The morphological examination of bone marrow biopsy was consistent with CML. His disease-spesific calculated risk scores were as follows; Sokal score [5]: 0.71 (low-risk), Hasford (Euro) score [6]: 77 (low-risk), and EUTOS score [7]: 61 (low-risk). He was treated with hydroxyurea (1.5 g/day) and allopurinol until the cytogenetic and molecular tests resulted.

Conventional cytogenetic analysis on the bone marrow detected 38-45,XY,t(11;22)(q23;q11.2)[5]/46,XY,t(11;22)(q23;q11.2), without the involvement of chromosome 9. However, the presence of a cryptic BCR/ABL fusion transcript was detected (BCR-ABL/G6PD: 0.268) by reverse transcription polymerase chain reaction (RT-PCR) on peripheral blood via using specific primers for b2a2, b3a2. The patient was diagnosed as having chronic phase CML in February 2013. Treatment with imatinib mesylate, a selective tyrosine kinase inhibitor (TKI), was started at a dose of 400 mg/day.

### First Line TKI (Imatinib mesylate) treatment

#### 1^st^ Month

He had no active symptoms and his spleen was non-palpabl in physical examination. A rapid complete hematologic response was achieved.

#### 3rd. Month

He had suffered from bilateral leg and ankle pain without limiting his daily activities. Fluorescence in situ hybridization (FISH) analysis performed on 200 metaphases and nuclei revealed no t(9;22), whereas conventional cytogenetics revealed 17% Ph chromosome positivity (major cytogenetic response). A control bone marrow biopsy revealed a normocellular marrow histology. BCR-ABL/G6PD ratio did rapidly decrease to 0.0135. Starting from this time point our molecular laboratory have been started to monitorise CML patients by quantitative RT-PCR on the International Scale (IS). BCR-ABL/ABL transcript level was 0.0471% according to the IS [8].

#### 10.5th. Month

Despite being in complete hematologic and cytogenetic remisson, his BCR-ABL/ABL transcript levels increased from 0.0471% to 1.4034%, indicating imatinib failure. A second generation TKI, nilotinib, was then started at a dose of 2 x 400 mg. The point mutations of T315I, F311L, and M351T mutations were all negative, which may be related to imatinib failure. JAK2V617F mutation was also found to be negative. The related donor search revealed a HLA-DRB1 mismatched (9/10 compatible) donor.

### Second Line TKI (Nilotinib) Treatment

**Figure 1:**
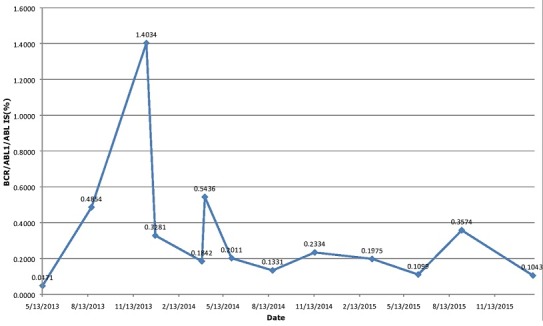
Molecular monitorisation of the patient by quantitative real time PCR on the International Scale (IS)

Under nilotinib treatment, IS ratios were 0.5436%, 0.2011%, 0.1331%, and 0.2334% at the 3, 6, 9, and 11 months of therapy, respectively. Conventional cytogenetic analysis at 11. month of therapy was consistent with a complete cytogenetic response as expected. The patient was advised to continue nilotinib. After 12 months of therapy, he had to be considered as warning (0.1% = BCR/ABL < 1%) according to the European LeukemiaNet 2013 recommendations [9]. His detailed RT-PCR results during extended follow-up are given in Figure 1. The patient is still on nilotinib therapy without any adverse events.

## Discussion

Variant Ph chromosome translocations are found in 2-10% of CML patients [1, 2]. It seems to effect any chromosome in which most susceptible breakpoints are; 1p36, 3p21, 5q31, 6p21, 9q22, 10q22, 11q13, 12p13, 17p13, 17q21, 17q25, 19q13, 21q22, 22q12, and 22q13 [1]. In simple variant translocations; the 22q11 segment is translocated on chromosome other than 9. In complex variant translocations; there is a third translocation, t(9;22;V), where “V” is a variable partner chromosome [3]. On the other hand, two possible mechanisms have been suggested for the formation of cryptic rearangements. First one is a “single event” via simultaneous breakage of several chromosomes followed by mismatched joining. The second one is a “multi-step mechanism” in which a classical Ph translocation is followed by further translocation events involving chromosome 9 and 22 and/or other chromosomes [10].

In a large series of early chronic phase CML patients treated with imatinib as a frontline therapy, 5% of the cases showed variant translocation. The main mechanism in the genesis of the variant translocation was one-step mechanism (75%), and the majority of the patients showed a 3-way pattern. The clinical characteristics and long-term outcomes of patients with variant Ph translocations were similar to those with classic Ph translocations [2, 11]. In a database 59 (6.1%) of the 974 patients were found to carry four-way Ph translocations [12]. Chromosome 7 and 8 were the most frequently involved chromosomes in this type of translocations, other than chromosome 9 and 22. Additionally, CML patients with variant t(5;9;10;22) and t(9;22;15;19) were reported from India and Japan, respectively [13, 14]. The latter case was effectively treated with nilotinib as was in our patient [14].

We presented a case with CML with t(11;22)(q23;q11.2) variant translocation and also this case was BCR/ABL positive. The breakpoint we identified, 11q23, is most commonly associated with childhood acute leukemias. To the best of our knowledge, he was the first CML case described from Turkey. We detected the BCR/ABL fusion by using RT-PCR method, so we could not detect the derivative chromosome on which the BCR/ABL fusion gene was located. It may be a simple variant translocation or a masked Ph translocation in which the fusion gene was located to der(22). A CML case was reported from Japan who had t(11;17)(q23;q21) not alone but additional to Ph chromosome [15]. In a study from Armenia; firstly, BCR/ABL fusion gene was observed in 100% nuclei by interphase FISH in three cases with t(2;22;9;22). After imatinib therapy, second cytogenetic investigations revealed that two clones were present: a Ph chromosome negative clone with t(2;22) and a complex translocation with the BCR/ABL gene on der(2). Notably t(2:22) was the primary chromosomal change, and probably, BCR/ABL was result of the translocation between der(2)t(2;22) and chromosome 9 [10]. Similar to these 3 cases, in the present case primary chromosomal change may be t(11;22) and BCR/ABL was result of translocation between der(11) and chromosome 9 which could not be detected by our conventional cytogenetic analysis. Or BCR/ABL fusion gene was located to der(22)t(11;22) termed as masked Ph.

JAK2 gene at chromosome 9p24 encodes for the JAK2 kinase inducing proliferative activity of hematopoietic cells in Philadelphia-negative myeloproliferative neoplasms. This mutation seems to have no prognostic significance in CML [16]. Albeit negative, we also searched for this mutation. As a limitation, we were not able to perform FISH analysis at the time of the diagnosis due to logistic limitations. The initial FISH analysis is not only essential for the correct diagnosis of CML, but it is also important for providing unique information about the action mechanism of the variant translocation(s) and/or the presence of a masked Ph [13, 17].

## Conclusion

In conclusion, BCR/ABL may be located on chromosomal band other than 22q11. PCR analysis was required, because conventional cytogenetic analysis alone could not demonstrate this event. This case points out the requirement of combining conventional cytogenetics and RT-PCR analysis (especially with IS) for the diagnosis of CML patients with variant translocation or masked Ph. TKIs are useful and effective in the medical treatment of these cases similar to those CML patients without variant translocation.

## Competing interests

The authors declare no competing interests.
